# Supporting the translation of multiscale research in rare disease

**DOI:** 10.1242/dmm.050495

**Published:** 2023-09-22

**Authors:** Kirsty M. Hooper, Julija Hmeljak

**Affiliations:** The Company of Biologists, Bidder Building, Station Road, Histon, Cambridge CB24 9LF, UK

## Abstract

**Summary:** In anticipation of our upcoming Special Issue, ‘Translating Multiscale Research in Rare Disease’, we celebrate the strides taken in rare disease research that are improving patient diagnosis, prognosis and treatment.

Since its launch in 2008, Disease Models & Mechanisms (DMM) has been a welcoming home for research in rare and ultra-rare disease. Owing to its position at the intersection between disease biology, human genetics and translational applications, our journal is uniquely suited to fostering advances in the rare disease research field. During DMM's first decade, preclinical and translational rare disease research benefitted from a number of landmark advances, from the increased accessibility of next-generation genome and transcriptome sequencing to powerful genome editing, to novel human stem cell-based modelling platforms. To highlight these achievements, we launched a dedicated subject collection (Hmeljak and Justice, 2019). We are delighted that, in the years since its launch, this subject collection has grown and evolved into a rich showcase of impactful advances in rare disease.

“Rare disorders in medicine are commonly overlooked, but when taken together, rare disorders become a formable group that should not be ignored.” Kate Rauen, Professor Emerita, Division of Genomic Medicine, Department of Pediatrics, University of California, Davis, USA and Guest Editor of DMM's upcoming Special Issue.

More-refined model systems with high predictive validities are crucial for promoting clinical advances in diagnosing and treating rare disease. Recent research published in DMM is firmly centred on achieving clinical benefit through insights from a broad range of validated models. For instance, research in slime mould (Huber, 2021), in human neurons derived from induced pluripotent stem cells (iPSCs) (Chear et al., 2022) and in a porcine model (Swier et al., 2023) has generated new insights into the pathogenesis of neuronal ceroid lipofuscinoses. Each of these model systems not only enriches our understanding of the accumulation of intracellular material in these disorders but also reveals novel therapeutic targets. Key progress has also been made in testing new therapeutic strategies for the ultra-rare sarcoma desmoplastic small round cell tumour, using patient-derived cell lines and patient-derived mouse xenograft models (Smith et al., 2022; Zuco et al., 2023), and a new mouse model of rhabdomyosarcoma better recapitulates the human disease to enable the exploration of a wider range of therapies (Nakahata et al., 2022). Rare muscular disorders are yet another area that has strongly benefitted from advanced model systems (Crawford et al., 2022; Fernández-Costa et al., 2023; van Putten, 2022). Probing the pathomechanisms of these diverse diseases across scales – in a range of laboratory systems and along a spectrum of biological manifestations – is essential to further our understanding of these diseases that, individually, affect so few people, but together have an enormous impact on human health.

“Not only is it important to understand mechanisms of rare disease, but it's important to push forward with treatments, as it is not uncommon that treatment for rare disorders sheds light on disease mechanisms and novel treatments for more common diseases and disorders. Understanding uncommon disorders sheds light and sparks novel ideas for treatment of the common disorders we see in medicine.” Kate Rauen.

Rare disease research most certainly enriches our understanding of common disorders, as well as of fundamental biological processes. Lafora disease is a rare and fatal epilepsy characterised by the deposition of overlong branched glycogen. A recent study deciphered the role of a glycogen-regulating protein, malin, in the pathogenesis of Lafora disease, which has provided insight into a glycogen metabolism mechanism that may also be involved in more common diseases, such as those involving insulin resistance (Mitra et al., 2023). Furthermore, a study of Paget's disease, a rare bone remodelling disorder, has also unveiled a novel regulator of bone metabolism that has relevance beyond this disorder (Wani et al., 2022). Osteogenesis imperfecta is another rare bone disorder that arises from dysregulated collagen. Clinical and animal modelling data have helped to dissect the variable phenotypes stemming from just a few recurrent mutations, which will inform clinical diagnosis and prognosis for patients (Garibaldi et al., 2022; Lee et al., 2022) and may also inform on more common bone disorders. Rare disease encompasses a wide breadth of disorders that affect almost every human biological process and organ. By interrogating the mechanisms of these diseases, we are blurring the boundaries between rare and common disorders that converge on fundamental biological functions.

Although the community has much to celebrate, these significant advances are also unearthing complex challenges, particularly in rare genetic disorders. Understanding the functional implications of causative genetic variants is incomplete owing to most genome-wide association study (GWAS) databases not including populations of diverse ancestries (Lek and Mardis, 2021; Wonkam, 2023). This is leading to further disparities in clinical outcomes within the rare disease community, as diagnoses and treatments are primarily tailored to patients of European ancestry (Lek, 2022). Furthermore, sophisticated ‘*n* of 1’ therapies based on individual patient variants currently remain out of reach for patients who lack the resources to fully engage with researchers. Efforts are being made to expand the diversity of participants in GWASs globally and to support research that is driven by scientists in the Global South. Owing to the paucity of knowledge for certain rare diseases, the patient community needs to be at the core of these ongoing and future research efforts. Engaging and establishing meaningful partnerships with patients and their advocates from diverse backgrounds will ensure that any clinical outcomes will benefit all patients.

As we consider these encouraging advances and persistent challenges, we are reassured that the community has much to look forward to. Translating fundamental research into meaningful clinical impact is of course a key goal for rare disease researchers. At DMM, one of our core aims is to support the translational potential of the high-quality, peer-reviewed rare disease research we publish. By launching our upcoming Special Issue (Box 1 and Fig. 1), we are amplifying our commitment to the rare disease community of researchers, clinicians, patients, and their carers and advocates.

**Box 1. DMM Special Issue on Translating Multiscale Research in Rare Disease**

DMM invites you to submit your latest research for our upcoming Special Issue, Translating Multiscale Research in Rare Disease. The issue is being driven by DMM Editors Monica Justice [The Hospital for Sick Children (SickKids), Toronto, Canada], Karen Liu (King's College London, UK) and Monkol Lek (Yale School of Medicine, USA), and Guest Editor Kate Rauen (University of California, Davis, USA). Research in various model systems can reveal rare disease mechanisms at genetic, molecular, cellular, tissue, organ and organism levels. DMM welcomes Research and Resource articles focused on the genomics, phenomics, networks, mechanisms and pathways of rare diseases, to foster meaningful clinical progress in their diagnosis and treatment.

The issue will also include specially commissioned Reviews, Perspectives and Interviews from leaders in the field and will be widely promoted online and at key global conferences, guaranteeing maximum exposure for your work. It is scheduled for publication in Spring 2024, although note that our continuous publication model means that DMM will publish your article as soon as it is ready; you will not have to wait for the rest of the issue to be complete. We look forward to receiving your breakthrough rare disease research by Monday 6 November 2023.

**Fig. 1.**
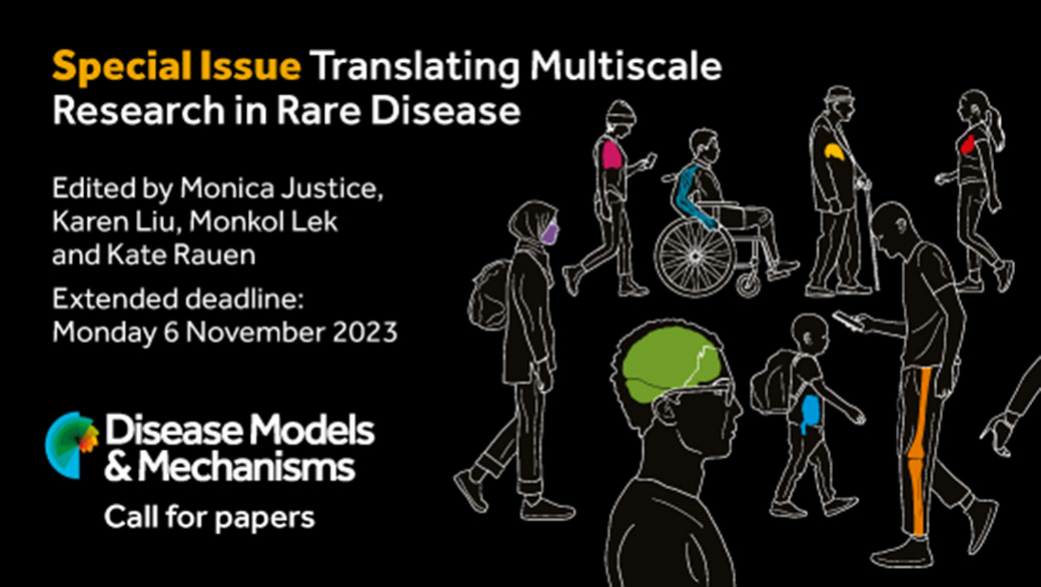
**Submit your research to our upcoming Special Issue, Translating Multiscale Research in Rare Disease, by Monday 6 November 2023.** This image is by neilsmithillustration.co.uk and published under the CC-BY 4.0 license for this article.
